# Effects of intravenous administration of allogenic bone marrow- and adipose tissue-derived mesenchymal stem cells on functional recovery and brain repair markers in experimental ischemic stroke

**DOI:** 10.1186/scrt159

**Published:** 2013-01-28

**Authors:** María Gutiérrez-Fernández, Berta Rodríguez-Frutos, Jaime Ramos-Cejudo, M Teresa Vallejo-Cremades, Blanca Fuentes, Sebastián Cerdán, Exuperio Díez-Tejedor

**Affiliations:** 1 Department of Neurology and Stroke Centre, Neuroscience and Cerebrovascular Research Laboratory, La Paz University Hospital, Neuroscience Area of IdiPAZ (Health Research Institute), Autónoma University of Madrid, Madrid, 28046, Spain; 2 Laboratory for Imaging and Spectroscopy by Magnetic Resonance LISMAR, Institute of Biomedical Research Alberto Sols, CSIC-UAM, Madrid, 28029, Spain

## Abstract

**Introduction:**

Stem cell therapy can promote good recovery from stroke. Several studies have demonstrated that mesenchymal stem cells (MSC) are safe and effective. However, more information regarding appropriate cell type is needed from animal model. This study was targeted at analyzing the effects in ischemic stroke of acute intravenous (i.v.) administration of allogenic bone marrow- (BM-MSC) and adipose-derived-stem cells (AD-MSC) on functional evaluation results and brain repair markers.

**Methods:**

Allogenic MSC (2 × 10^6 ^cells) were administered intravenously 30 minutes after permanent middle cerebral artery occlusion (pMCAO) to rats. Infarct volume and cell migration and implantation were analyzed by magnetic resonance imaging (MRI) and immunohistochemistry. Function was evaluated by the Rogers and rotarod tests, and cell proliferation and cell-death were also determined. Brain repair markers were analyzed by confocal microscopy and confirmed by western blot.

**Results:**

Compared to infarct group, function had significantly improved at 24 h and continued at 14 d after i.v. administration of either BM-MSC or AD-MSC. No reduction in infarct volume or any migration/implantation of cells into the damaged brain were observed. Nevertheless, cell death was reduced and cellular proliferation significantly increased in both treatment groups with respect to the infarct group. At 14 d after MSC administration vascular endothelial growth factor (VEGF), synaptophysin (SYP), oligodendrocyte (Olig-2) and neurofilament (NF) levels were significantly increased while those of glial fiibrillary acid protein (GFAP) were decreased.

**Conclusions:**

i.v. administration of allogenic MSC - whether BM-MSC or AD-MSC, in pMCAO infarct was associated with good functional recovery, and reductions in cell death as well as increases in cellular proliferation, neurogenesis, oligodendrogenesis, synaptogenesis and angiogenesis markers at 14 days post-infarct.

## Introduction

Stem cell therapy offers promising results for stroke patients but its efficacy has not yet been confirmed in the first clinical trials [1]. Important recommendations concerning the time of administration, the best routes of administration and cell sources as well as the use of different experimental animal models prior to starting clinical trials have been made in recent years to better identify the best options for stem cell therapy in stroke patients [2-4].

Although the optimum administration time is unclear [5] and previous reports have focused on post acute-phase intervention [6], promising experimental animal data suggest that early stem cell administration can interrupt the initiation of the very beginning of the ischemic cascade [7,8]. In regard to the type of administration, experimental animal studies have shown autologous administration to be safe and effective [9]. The main limitation is that stem cell preparation takes time, so autologous cells can only be administered several weeks after an unexpected stroke. Nevertheless it is important to act in the acute phase, so allogenic administration may be a good alternative. In many experimental animal studies, the acute use of cells from the same species has been shown to be safe and has not produced rejection [10].

It was originally thought that stem cells needed to be placed within the damaged sites of the brain after ischemia in order to promote recovery. From this perspective putative administration routes have been focused on intra-arterial [11], intrastriatal [12], intracerebral [13] or intraventricular alternatives [14]. However, the possibility that the cells need not be in the brain itself for recovery to be achieved would allow the use of less invasive methods, such as intravenous (i.v.) administration [8].

In any case, one of the main questions is to identify the best source for stem cells that can be used after stroke, and this needs to be addressed by comparative studies under different conditions. The different stem cell types to be tested include embryonic, hematopoietic, neural and mesenchymal stem cells (MSC) [7,15-17]. The most promising of these are MSC since they can be derived from bone marrow (BM-MSC), or adipose tissue (AD-MSC). Both tissues are very abundant, easy to obtain and do not pose logistic or ethical problems [6,9,12]. In a recent comparative study of transitory ischemia in mice, i.v. administration of either BM-MSC or AD-MSC promoted recovery [18]. That study observed higher levels of angiopoietin-1 and hepatocyte growth factor (HGF) in the AD-MSC group compared with those receiving BM-MSC at 24 h after i.v. cell administration but it only employed MSC at 24 h and 48 h after transitory ischemia. We have previously shown that administration of allogenic BM-MSC promotes functional recovery and also augments expression levels of vascular endothelial growth factor (VEGF) in the brain after permanent middle cerebral artery occlusion (pMCAO) in adult rats [8]. However, the effects of allogenic BM-MSC and AD-MSC administration on functional recovery and the levels of brain repair markers associated with neurogenesis, oligodendrogenesis, synaptogenesis and angiogenesis at 14 d have not yet been examined in the pMCAO model.

We hypothesize that acute i.v. administration of allogenic AD-MSC would be as effective as that of allogenic BM-MSC after pMCAO in adult rats.

The present study compared the therapeutic potential of MSC from both cell sources for improving functional recovery, decreasing tissue damage and increasing brain repair associated markers at 14 d after pMCAO in rats.

## Materials and methods

### Animal ethics

The procedure was carried out at our Cerebrovascular and Neuroscience Research Laboratory, La Paz University Hospital, Madrid, Spain. All experiments were performed in compliance with our medical school's Ethical Committee for the Care and Use of Animals in Research. The experiments were designed to use the smallest number of animals and to minimize their suffering in accordance with the ethical standards of the Helsinki Declaration of 1975. The investigators responsible for functional evaluation and the molecular and histological studies were blinded to the treatment groups.

### Study design

#### Isolation of mesenchymal stem cells

Cultures were made of BM-MSC obtained from the tibia and femur of adult female Sprague-Dawley (250-300 g) rats that had been sacrificed by cardiac injection of potassium chloride (0.5 ml) as described previously [8]. First, both bones were placed in alcohol for 10 minutes and then in Hank's (1X) balanced salt solution (HBSS, Gibco, Paisley, UK) for 30 minutes. The bones were cut and bone marrow removed, 10 ml of this tissue was placed in 50 ml of DMEM 1X solution (DMEM Glu/Pyr, Gibco) with 75 μl penicillin/streptomycin (Sigma-Aldrich, St Louis, MO, USA) and 20% FBS (PAA Laboratories, GmbH, Pasching, Austria). Next, cells were washed and centrifuged twice at 390 g for 8 minutes at room temperature. The cells were placed in 75-cm^2 ^flasks (Nunc, Roskilde, Denmark)) and incubated at 37°C in 5% CO2 for 3 to 4 weeks. The culture medium was replaced approximately every 3 days. When cells reached 80 to 90% confluence, they were trypsinized using Trypsin-EDTA 0. 05% (Gibco) and expanded in another 75-cm^2 ^flask. On the third pass they were trypsinized and counted before being administered to the experimental animals.

Lipoaspirates from adult female Sprague-Dawley (250 to 300 g) were washed with sterile PBS and digested with an equal volume of 0.075% type I collagenase (Sigma-Aldrich,). The filtered cells were centrifuged at 390 g for 10 minutes and contaminating erythrocytes were removed to isolate the stromal vascular fraction (SVF). On the third pass they were trypsinized and counted before being administered to the experimental animals.

#### Characterization of MSC

At the time the cells were obtained, the cultures were characterized to confirm the presence or absence of MSC surface markers using the flow cytometric technique and analyzed with fluorescence-activated cell sorting (FACS). The cells were incubated for 20 minutes at 4°C in the dark with the following antibodies: CD90-fluorescein isothiocyanate (FITC) (AbD Serotec, Oxford, UK), CD29-Phycoerythrin (PE) (AbD Serotec), CD45-PE (AbD Serotec) and CD11b-PE (AbD Serotec). Matched isotype controls were purchased from Biolegend (Biolegend, San Diego, CA, USA). At least 1 × 10^4 ^cells per sample were acquired and analyzed.

#### Subjects

Subjects were adult male Sprague-Dawley rats, with an average body weight range of 250 to 320 g (Harlan Iberica SL, Barcelona, Spain). Animals were housed with free access to food and water at a room temperature of 21 ± SD 2°C, relative humidity of 45 ± 15% and a light/dark cycle of 12 h (7:00 to 19:00).

#### Experimental groups

The animals were randomly assigned to one of four experimental groups with 10 animals in each study group: group 1, the sham-operated group, underwent surgery without infarct and received a saline solution via the femoral vein; group 2, the iInfarct group, underwent surgery with permanent middle cerebral artery occlusion (pMCAO) and received the saline infusion via the femoral vein; group 3, the BM-MSC group, underwent pMCAO surgery and received a BM-MSC infusion via the femoral vein; and group 4, the AD-MSC group, underwent pMCAO surgery and received an AD-MSC infusion via the femoral vein.

#### Surgical procedure

Anesthesia was induced by intraperitoneal injection of a solution of ketamine (25 mg/kg), diazepam (2 mg/kg), and atropine (0.1 mg/kg) at a dose of 2.5 ml/kg. Analgesia was provided by meloxicam 2 mg/kg by a subcutaneous route. A small craniectomy was made above the rhinal fissure over the branch of the right middle cerebral artery (MCA). The MCA branch was permanently ligated just before its bifurcation into the frontal and parietal branches with a 9-0 suture. Both common carotid arteries were then occluded for 60 minutes as previously described [8].

#### Physiological monitoring

In all animals, the femoral artery was cannulated during surgery and ischemia, to allow continuous monitoring of physiological parameters (glycemia, blood gases and blood pressure) (Monitor Omicron ALTEA RGB medical devices, Madrid, Spain). Cranial and body temperature were also monitored and maintained at 36.5 ± 0.5°C.

#### Cell administration

Intravenous injections of 2 × 10^6 ^MSC in 650 μl saline were administered over 4 minutes through the femoral vein. Infarct animals underwent cerebral ischemia as in the treated animals but received only a saline infusion through the femoral vein. Sham-operated animals received the saline infusion through the femoral vein but did not undergo cerebral ischemia. The sham-operated and infarct groups both received a single 650 μl saline infusion without MSCs over 4 minutes. Either the saline or MSC solution was administered in the acute phase 30 minutes after common carotid artery reperfusion. The route, dose and timing of administration have been used in a previous study [8].

### *In vivo *analyses

#### Functional evaluation scales

In all animals functional evaluation scales were performed at baseline and at 24 h and 14 d after surgery. All rats were evaluated using a variant of the Rogers scale [8,19-21] and the rotarod test. The Rogers scale scores functional status as follows: no deficit (0); failure to extend left forepaw (1); decreased grip of the left forelimb when the tail is pulled (2); spontaneous movement in all directions, contralateral circling if pulled (3); circling or walking to the left (4); movement only when stimulated (5); unresponsive to stimulation (6); and dead (7). The rotarod test was used to evaluate the motor performance of the rats. Beginning three days before pMCAO, rats were trained on an accelerating (4 to 40 rpm) rotarod. All animals received a 3-day training program consisting of three sessions per day, and the time each animal remained on the rotarod was measured. Before surgery on the experimental day, the time spent moving on the rotarod without falling was measured twice per animal with a 15-minutes interval between each trial. The mean of the two trials was calculated for each rat [22].

#### Migration and implantation of stem cells by magnetic resonance imaging (MRI)

MSC were magnetically labeled using Endorem™ (superparamagnetic iron oxide). (Guerbet, Roissy CdG Cedex, France). Both migration and implantation of the stem cells were analyzed at 24 hours and 14 d by MRI with T2 maps (flash sequence). Endorem™(Guerbet)-labeled MSC were transplanted into five animals in each group.

#### Measurement of volume of ischemic lesion by MRI

Lesion volume was analyzed at 24 h and 14 days after surgery by MRI (Bruker Pharmascan, Ettlingen, Germany), (7 Tesla horizontal bore magnets) using T2 maps (RARE 8 T2, 180° flip angle, three averages). Ten contiguous coronal slices (thickness, 1 mm) were acquired with a field of view of 35 × 35 mm and a matrix size of 256 × 256 (repetition time (TR) 3000 ms, echo time (TE) 29.5 ms, imaging time 25.5 minutes, three averages). All images were processed using the J 1.42 Image program (NIH software, Bethesda, MD, USA). After contrast adjustment, the contours of the hemispheres were traced manually on each slice. The infarct volumes were estimated by integrating the partial measurements derived from the cross-sectional areas and the distance between sections. To correct for the brain edema effect, lesion volume was determined by an indirect method:

Infarct area = (Area of the intact contralateral hemisphere) - (Area of the intact ipsilateral hemisphere) [23].

Then lesion volume was expressed as a percentage of the intact contralateral hemispheric volume.

### Post-mortem analyses

#### Migration and implantation of stem cells by DiI

MSC were labeled with DiI (Celltracker CM-DiI, Molecular Probes ™, Eugene, Oregon, USA) prior to administration and then, migration and implantation were analyzed at 14 d post-administration using immunofluorescence. The DiI (Molecular Probes ™)-labeled MSC were administered into five animals in each treatment group.

#### Measurement of volume of ischemic lesion by H&E

Lesion size was estimated with H&E staining of brain sections at 14 d. Infarction volume was expressed as the percentage of brain tissue affected by ischemia in the right hemisphere as evaluated on 10-μm-thick sections. Brains were sectioned at the optic chiasma and at the infundibular stalk. The resultant brain block from between these two cuts was placed in 4% paraformaldehyde for 24 h and 30% sacarose PBS buffer for 3 days. Optimal cutting temperature (OCT)-embedded samples were coronally sectioned in 10-μm-thick slices. Every twentieth slice, that is, a total of four of these slices (numbers 1, 21, 41 and 61), separated from each other by 100 μm, were stained with H&E, which reveals an ischemic area as a well-defined pale region. A digitized image was made of these slices (Epson Perfection 1260 scanner, Suwa, Nagano, Japan) and used to automatically measure the ischemic area (Image Pro plus 4.0, Media Cybernetics, Rockville, MD, USA) [8,20,24]. Lesion volume was determined using the method described in MRI measurement of volume of ischemic lesion.

#### Cell death

Apoptotic cell death was detected by biotin-dUTP nick end-labeling mediated by terminal deoxynucleotidyl transferase (TUNEL) staining, using TdT-FragEL DNA Fragmentation Detection Kit, Oncogene Research Products, San Diego, CA, USA) following the methodology indicated by the manufacturer. We chose a single rostral-caudal coronal section per animal and counted the number of apoptotic cells in the peri-infarct zone using a 40× objective on the optic microscope (Olympus, BX41, Olympus Corporation, Tokyo, Japan) and image analysis software (Image-Pro Plus 4.1, Media Cybernetics, Rockville, MD, USA). Cells undergoing apoptosis were identified based on their nuclear morphology and dark color [8,20].

#### Immunohistochemistry

All animals were given 50 mg/kg of daily intraperitoneal BrdU (Sigma-Aldrich) on days 4 to 7 after ischemia. This administration protocol was based on previous reports that proliferation peaked 4 to 10 days after injury [25,26]. Animals were sacrificed 14 d after surgery by transcardial perfusion and decapitation. Their brains were fixed and stored at 4°C and the following day the tissue was placed in cryoprotectant solution at -80°C. Serial coronal sections were cut at 10 μm on a cryostat (LEICA CM1950, Leica Microsystems, Heilderbeg, Germany) and later studied by immunohistochemistry for cellular proliferation. Brain sections were treated with BrdU In-Situ Detection kit (BD Biosciences, Franklin Lakes, NJ, USA).

#### Immunofluorescence

The sections were studied using different immunofluorescent antibodies as follows: the neuronal markers, neuronal nuclei (NeuN) (monoclonal antibody diluted 1:100, Millipore, Billerica, MA, USA) and neurofilament (NF) (monoclonal antibody diluted 1:100, DAKO, Denmark A/S, Glostrup, Denmark); the astrocyte marker, glial fiibrillary acid protein (GFAP) (monoclonal antibody diluted 1:400, Chemicon, Temecula, CA, USA); the vascular endothelial growth factor (VEGF) marker (polyclonal antibody diluted 1:500, Millipore); the oligodendrocyte (Olig-2) marker (polyclonal antibody diluted 1:500, Millipore); the synaptogenesis marker, synaptophysin (monoclonal antibody diluted 1:200, Sigma-Aldrich); and the brain-derived neurotrophic factor (BDNF) (polyclonal antibody diluted 1:1000, Millipore), followed by goat anti-mouse Alexa Fluor 488 and anti-rabbit Alexa Fluor 594 (1: 750, Molecular Probes, Invitrogen, Barcelona, Spain). Also, we used BrdU (monoclonal antibody diluted 1:50, DAKO) followed by goat anti-mouse Alexa Fluor 594 as a proliferation marker. All sections were mounted with H-1200 VectaShield mounting medium for fluorescence with diamidino-2-phenylindole (DAPI, Vector, Atom, Alicante, Spain). Samples were examined using a LEICA TCS SPE spectral confocal microscope (Leica Microsystems, Heidelberg, Germany) and the confocal images were analyzed using LEICA software LAS AF, version 2.0.1 Build 2043. The images were acquired as a confocal maximum projection.

#### Western blot

Proteins were isolated from peri-infarct tissue and their concentrations determined using a BCA protein assay kit (Pierce, Rockford, IL, USA). Twenty micrograms of protein were loaded onto 10% acrylamide SDS-gels. Following electrophoresis at 100 V for 1 h, protein was transferred to polyvinylidene fluoride (PVDF) membranes (Bio-Rad Laboratories, Hercules, CA, USA). Membranes were blocked in 5% fat-free dry milk dissolved in Tris-buffered saline pH 8.0 (TBS) plus 0.1% Tween-20 (TBS-T) for 1 h and probed overnight at 4°C with the following antibodies at the designated dilutions: NF (monoclonal antibody diluted 1:100, DAKO); GFAP (monoclonal antibody diluted 1:400, Chemicon); VEGF (polyclonal antibody diluted 1:500, Millipore); Olig-2 (polyclonal antibody diluted 1:500, Millipore); synaptophysin (monoclonal antibody diluted 1:200, Sigma-Aldrich); BDNF (polyclonal antibody diluted 1:1000, Millipore) and β-actin (monoclonal antibody diluted 1:400, Sigma-Aldrich), which was used as a control protein. After rinsing with 0.5% TBS-T solution, the membranes were incubated with the secondary antibody, a donkey anti-rabbit and anti-mouse antibody conjugated with horseradish peroxidase (HRP, Chemicon) for 1 h at room temperature. Signals were detected by enhanced chemiluminescence (ECL, GE, Healthcare Europe GmbH, Freiburg, Germany) before exposure on radiographic film. The density of stained bands was scanned and quantified by the Scion Image and 1-D Manager Version 2.1 (Scion Corporation, Frederick, Maryland, USA). To reduce differences between animals, at least three western blots were performed for each time point and animal. In addition, at least two or three repeated samples were always included in every set of experimental samples as internal standards.

#### Statistical analysis

Quantitative data are shown as mean values ± SD. The Kruskal-Wallis test followed by the Mann-Whitney test were used to compare the functional evaluation score, lesion size, cell death and number of BrdU-positive cells, while VEGF, BDNF, SYP, Olig-2, NF and GFAP levels were compared between animals receiving MSC from either cell source among the various groups. Values of *P *< 0.05 were considered significant at a 95% CI; we used statistical software SPSS 16 for Windows for analysis.

## Results

Physiological parameters remained within normal limits throughout the procedure and showed no significant differences between groups.

### Characterization of MSCs

**Figure 1:**
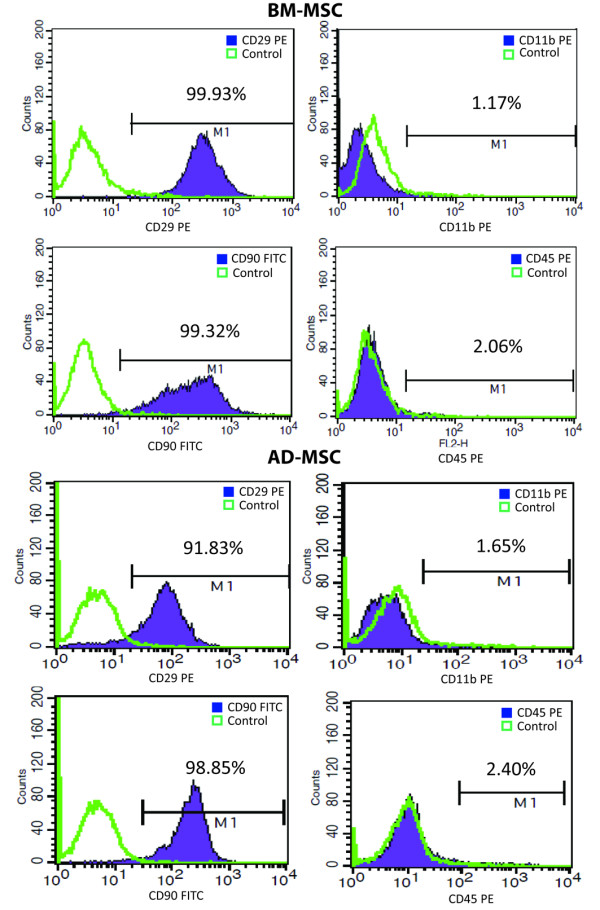
**Flow cytometry characterization of bone marrow-derived mesenchymal (BM-MSC) and adipose tissue-derived mesenchymal (AD-MSC) cells before administration**. Both BM-MSC (up) and AD-MSC (down) were analyzed before administration. MSC expressed markers CD29 and CD90 (left) and were negative for CD45 and CD11b. Both BM-MSC and AD-MSC showed similar results.

We confirmed that CD90-FITC (AbD Serotec) and CD29-PE (AbD Serotec) surface markers were positive in MSC, whereas the CD45-PE (AbD Serotec) and CD11b-PE (AbD Serotec) surface markers were negative (Figure 1). The MSC showed a typical fibroblast-like cell morphology.

### Allogenic i.v. administration of BM-MSC and AD-MSC improves functional recovery after pMCAO

**Figure 2:**
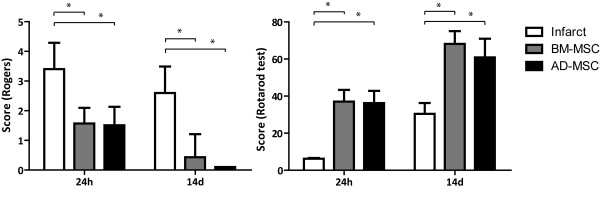
**Acute intravenous (i.v.) administration of allogenic bone marrow-derived mesenchymal (BM-MSC) and adipose tissue-derived mesenchymal (AD-MSC) cells improved functional recovery at 24 h and 14 d after permanent middle cerebral artery occlusion (pMCAO)**. The scores in the Rogers (left) and rotarod tests (right) are shown. Both treatments significantly improved functional recovery with no differences in recovery between the two groups (BM-MSC or AD-MSC). Data are expressed as mean ± SD (*n *= 10); *P < 0.05.

Animals were clinically evaluated at 24 h and 14 d and the corresponding functional score obtained for each group (Figure 2). The sham-operated group did not show functional deficit. On both the Rogers and the rotarod test, the treated groups showed good functional recovery regardless of cell source, bone marrow or adipose tissue, without significant differences in functional scores between treated groups at either time point. In the Rogers test, the BM-MSC (1.57 ± 0.53; 0.43 ± 0.78) and AD-MSC (1.51 ± 0.62; 0 ± 0) groups had significantly improved functional recovery compared with the untreated infarct group (3.4 ± 0.89; 2.6 ± 0.89) at 24 h and at 14 d, respectively (*P *< 0.05). In the rotarod test the BM-MSC (37 seconds ± 6.36 seconds; 68.15 seconds ± 6.86 seconds) and the AD-MSC (36.2 seconds ± 6.63 seconds; 60.9 seconds ± 10.04 seconds) groups had significantly improved functional recovery compared with the infarct group (6.25 seconds ± 0.35 seconds; 30.45 seconds ± 5.87 seconds) at 24 h and at 14 d, respectively (*P *< 0.05).

### Migration and implantation of MSC was not observed via i.v. administration

Migration and implantation of MSC were studied by MRI and DiI. Labeled MSC were not observed in the infarct group because they were not administered to this group. Endorem-labeled and DiI-labeled MSC were only injected into the treated groups. However, neither migration nor implantation was observed on either MRI or immunofluorescence images in the injured area after i.v. administration of either BM-MSC or AD-MSC.

### Infarct size after pMCAO measured by MRI and H&E does not change after systemic MSC administration

**Figure 3:**
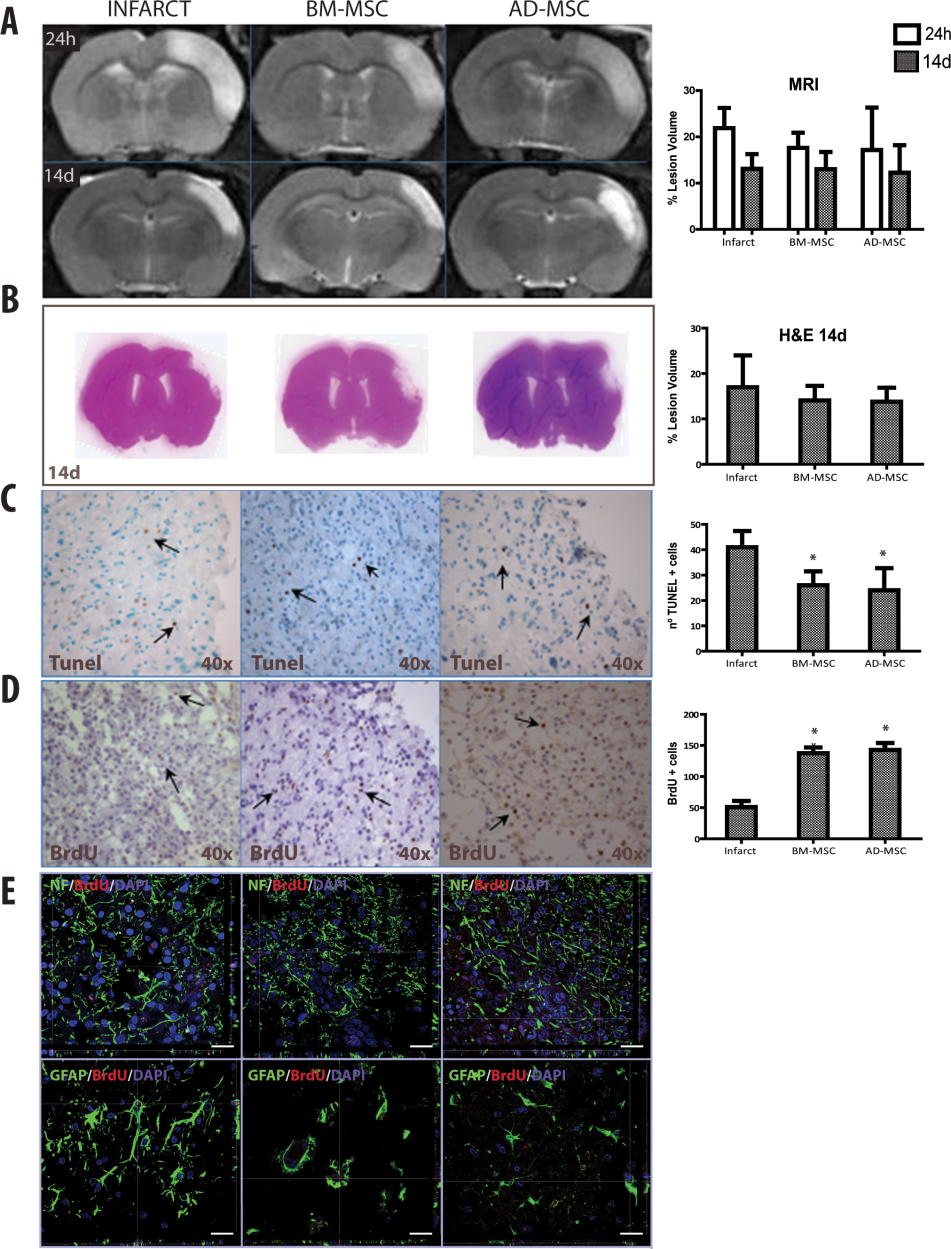
**Effects of acute and allogenic intravenous (i.v.) administration of bone marrow-derived mesenchymal (BM-MSC) and adipose tissue-derived mesenchymal (AD-MSC) cells on infarct volume, cell death and cell proliferation after permanent middle cerebral artery occlusion (pMCAO)**. (**A**) Magnetic resonance imaging (MRI) showed that infarct volume was not significantly decreased after BM-MSC or AD-MSC administration. (**B**) H&E staining showed infarct volume was not diminished at 14 d. (**C**) TUNEL staining at 14 d showed cell death was diminished after BM-MSC and AD-MSC administration. (**D**) Cell proliferation detected by bromodeoxyuridine (BrdU) staining at 14 d was increased in the BM-MSC and AD-MSC groups. (**E**) BrdU co-labeling with glial fiibrillary acid protein (GFAP) and neurofilament (NF) in the infarct, BM-MSC and AD-MSC groups in the peri-infarct zone at 14 days (scale bars = 20 μm). Data are expressed as mean ± SD (*n *= 10); *P < 0.05.

Lesion size at 24 h and 14 d after the experimental procedure was evaluated on MRI (Figure 3A). All values were lower at 14 d than at 24 h because the edema, which appeared in the first few days after ischemia, had decreased after two weeks. Neither of the treatments, BM-MSC (17.65% ± 3.27%; 13.04% ± 3.69%) or AD-MSC (17.16% ± 9.19%; 12.25% ± 5.97%), reduced lesion volume significantly with respect to the infarct group (21.89% ± 4.38%; 13.12% ± 3.16%) at either time point on MRI. Also, the H&E staining at 14 d showed that neither of the treatments, BM-MSC (14.09% ± 3.19%) or AD-MSC (13.73% ± 3.20%), had decreased lesion volume significantly with respect to the infarct group (16.98% ± 7.03%) (Figure 3B).

### Intravenous administration of allogenic MSC decreased cell death after pMCAO

Sham-operated animals did not show TUNEL + cells. There were no TUNEL+ cells in the contralateral hemisphere in any operated animal. At 14 d, the infarct group (41 ± 6.4) showed significantly more TUNEL+ cells than the BM-MSC (26 ± 5.5) and AD-MSC (24 ± 8.79) groups in the peri-infarct zone. In conclusion, both treatments significantly reduced the number of TUNEL+ cells compared to the number in the infarct group at 14 d (*P *< 0.05) (Figure 3C).

### Intravenous administration of allogenic MSC increased cellular proliferation after pMCAO

Sham-operated animals did not present BrdU-positive cells in the peri-infarct lesions. Quantitative analysis of BrdU-positive cells showed that the infarct group (51 ± 9.9) displayed a significantly smaller decrease in the number of BrdU-positive cells in the peri-infarct zone at 14 d after focal cerebral ischemia than did the BM-MSC (137.5 ± 9.2) or AD-MSC (142.81 ± 11.12) groups (Figure 3D). At 14 d, we observed BrdU co-labeling with GFAP and NF in the infarct, BM-MSC and the AD-MSC groups in the peri-infarct zone (Figure 3E).

### Intravenous administration of allogenic MSC modified brain repair markers levels after pMCAO

**Figure 4:**
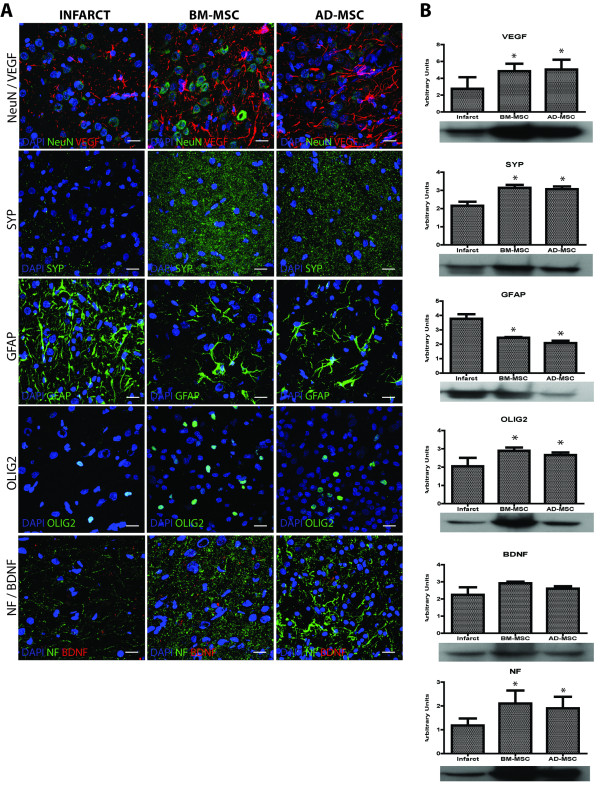
**Expression of brain repair markers in the peri-infarct area at 14 d after acute intravenous (i.v.) administration of allogenic bone marrow-derived mesenchymal (BM-MSC) and adipose tissue-derived mesenchymal (AD-MSC) cells in the permanent middle cerebral artery occlusion (pMCAO) model**. (**A**) Confocal images showing the levels of vascular epithelial growth factor (VEGF), neuronal nuclei (NeuN), synaptophysin (SYP), glial fiibrillary acid protein (GFAP), oligodendrocyte (OLIG2), brain-derived neurotrophic factor (BDNF) and neurofilament (NF) at 14 d in the infarct area after BM-MSC and AD-MSC administration (scale bars = 20 μm). (**B**) Western blot analysis showing that levels of VEGF, SYP, OLIG2, NF and BDNF were augmented at 14 d after BM-MSC and AD-MSC administration. The levels of GFAP were decreased in both groups. There were no significant differences in the results with respect to the cell source. Data are shown as mean ± SD (*n *= 3);*P < 0.05.

At 14 d after pMCAO the levels of brain repair markers were analyzed in confocal microscopy and confirmed by the western blot protein levels as arbitrary units (A.U.) (Figure 4). Compared with the infarct group (2.75 (A.U.) ± 1.38 (A.U.)), VEGF levels were significantly higher in the rat brain after administration of either BM-MSC (4.84 (A.U.) ± 0.89 (A.U.)) or AD-MSC (5.05 (A.U.) ± 1.16 (A.U.)). The levels of Olig-2 labeling were also significantly higher than in the infarct group (2.05 (A.U.) ± 0.46 (A.U.)) after the BM-MSC (2.89 (A.U.) ± 0.17 (A.U.)) and the AD-MSC (2.66 (A.U.) ± 0.14 (A.U.) treatments. Compared with the infarct group (2.15 (A.U.) ± 0.22 (A.U.), SYP levels were also significantly increased after BM-MSC (3.14 (A.U.) ± 0.16 (A.U.) and AD-MSC (3.06 (A.U.) ± 0.15 (A.U.) administration. Lastly, NF levels were significantly increased after either the BM-MSC (2.10 (A.U.) ± 0.54 (A.U.) or the AD-MSC (1.9 (A.U.) ± 0.48 (A.U.) treatment compared with the infarct group (1.18 (A.U.) ± 0.29 (A.U.). The BDNF levels were also higher after stem cell administration, but this increase did not reach statistical significance. The GFAP levels were significantly decreased after treatment with either BM-MSC (2.45 (A.U.) ± 0.05 (A.U.) or AD-MSC (2.075 (A.U.) ± 0.175 (A.U.) in comparison with the infarct group (3.76 (A.U.) ± 0.315 (A.U.). We did not observe any significant differences in brain repair markers between the BM-MSC- or AD-MSC-treated groups.

## Discussion

In this study we investigated the effects of acute i.v. administration of allogenic BM-MSC and AD-MSC after permanent focal ischemia in rats. We found that BM-MSC and AD-MSC i.v. administration significantly improved functional recovery despite the lack of a reduction in infarct volume, or the apparent absence of migration or implantation by the administered MSC to the damaged brain. Animals receiving MSC from either source showed decreased cell death and increased cell proliferation, VEGF, Olig-2, SYP and NF levels as well as reduced GFAP levels in the brain 14 d after pMCAO.

Importantly, in this study of acute administration of allogenic cells there were no differences in the effects of the treatment between the cells from either source, bone marrow or adipose tissue, that might have clinical implications for the translation of this treatment to human trials.

### BM-MSC and AD-MSC administration improves functional recovery independently of infarct volume and cell migration/implantation

The significant improvement in functional scales at 24 h was even more notable after 14 d of pMCAO, and there were no significant differences between the effects of cells from either source (BM-MSC or AD-MSC). In a recent comparative study of transitory ischemia in mice, the authors reported better results when administering AD-MSC than when administering BM-MSC [18]. That study also reported a reduction in infarct volume that we did not find after BM-MSC or AD-MSC administration; these differences might be explained by the fact that we did not recanalize the MCA. In our study, migration and/or implantation of cells administered i.v. was not found in the peri-infarct area since we could not detect any trace of Endorem™ or DiI staining at either 24 h or 14 d. Previous studies observed that after i.v. administration, stem cells are detected in the lungs, liver, spleen and other organs [27-29]. Therefore, it might not be necessary for stem cells to migrate and graft onto the lesion site in order to obtain a positive functional result [8], due to the paracrine effects of MSC. This good outcome could be a consequence of the secretion of several growth factors that could act to enhance endogenous repair mechanisms normally activated in the brain after stroke such as endogenous neurogenesis, immunomodulation, brain repair and plasticity [8,17,30,31].

### BM-MSC and AD-MSC effects on cell death and cell proliferation

Tissues were better-conserved in animals treated with BM-MSC and AD-MSC. TUNEL marking was reduced and cellular proliferation (BrdU-positive cells) increased in the peri-infarct zone, compared with the infarct group at 14 d. These results agree with previous studies that reported reductions in apoptotic cell number and increases in cellular proliferation after AD-MSC [9] and BM-MSC administration [8,32]. In line with earlier studies, other reports have demonstrated that administration of MSC promotes cellular proliferation in the subventricular zone [32] and cell differentiation into neuroblasts in the peri-infarct area compared to infarct group [33].

### BM-MSC and AD-MSC raise brain repair markers levels in the ischemic brain

It is thought that MSC secrete a wide array of neurotrophins, growth factors, cytokines and other soluble factors [34] such as VEGF or BDNF, in response to repair processes, and this could amplify trophic factor levels in the brain [35]. Studies *in vitro *and *in vivo *demonstrate that such factors can promote cell proliferation, survival and differentiation [36], and consequently raise the possibility that stem cell administration could promote recovery by interfering with trophic factor signaling to modulate brain repair response. In this study we have found that the expression of neurogenesis, synaptogenesis, angiogenesis and oligodendrogenesis markers such as NF, Olig-2, SYP and VEGF was significantly augmented at 14 d after acute and allogenic BM-MSC and AD-MSC administration compared with the infarct group. A previous study by our group found augmented VEGF levels after BM-MSC administration [8]. However the authors of a recent comparative study did not find significant differences in brain VEGF levels after the administration of either AD-MSC or BM-MSC 24 h after transitory ischemia in mice [18]. We did not observe differences for this marker at 14 d in our current study either.

Recovery after stroke is a dynamic process [37] and the growth and trophic factors produced by MSC may affect synaptogenesis in the ischemic brain [38]. In our comparative study, we observed that SYP levels in the peri-infarct zone were significantly increased after BM-MSC and AD-MSC administration in comparison with the infarct group. It is known that levels of NF expression are increased 7 d after focal cerebral ischemia in the peri-infarct zone [39]. In our study we observed significantly elevated levels of this marker 14 d after MSC administration. Also, oligodendrocytes are sensitive to ischemic damage but in several previous stroke studies BM-MSC administration enhanced oligodendrogenesis and remyelinization [40,41]. In our comparative study we detected augmented levels of Olig-2 at 14 d. Another factor, BDNF, has been shown to be augmented after i.v. MSC administration [42] and we detected in our study that it was higher (but not significantly) in the treated groups than in the infarct group. Finally, we detected a reduction in the glial marker, GFAP, at 14 d after both BM-MSC and AD-MSC administration (without significant differences between either cell type), probably reflecting a reduction in glial scar formation as previously observed [40] and in agreement with a previous report on AD-MSC administration [9].

### Future landmarks for clinical translation

The intrinsic complexity of stroke and the Stem Cell Therapies as an Emerging Paradigm in Stroke (STEPS) criteria for translational research underline the importance of further studies that can compare different experimental conditions and different experimental animal models. From the viewpoint of clinical translation allogenic stem cells are attractive because they can be easily obtained from young healthy donors, amplified, and stored for immediate use when needed after a stroke. Furthermore, it is known that MSC do not express MCH-II, minimizing the risk of rejection in patients [43] and the use of cells from the same species during the acute phase has been shown to be safe and effective without signs of rejection in many different experimental animal studies [8,11,44]. As an administration route in a clinical setting we considered the i.v. route, which has already shown efficacy in previous studies, to be less invasive than others, such as intra-arterial or intracerebral routes [7-9]. Future comparative studies are needed to determine cell fate after i.v. administration, the mechanism of action and the best dose to be used in patients to eliminate the possibility of undesired side effects.

## Conclusions

In our comparative study, following pMCAO we showed that AD-MSC are as effective as BM-MSC in promoting recovery and increasing the levels of brain protection and repair markers after the experimental ischemic stroke. Both cell sources are ethically acceptable with adipose-derived cells being particularly abundant and easy to obtain without invasive surgery.

## Abbreviations

AD-MSC: adipose tissue-derived mesenchymal stem cells; ANOVA: analysis of variance; A.U.: arbitrary units; BCA: bicinchoninic acid; BDNF: brain derived neurotrophic factor; BM-MSC: bone marrow-derived mesenchymal stem cells; BrdU: bromodeoxyuridine; DAPI: diamidino-2-phenylindole; DMEM: Dulbecco's modified Eagle's medium; ECL: enhanced chemiluminescence; EDTA: ethylenediamine tetraacetic acid anticoagulant; FACS: fluorescence-activated cell sorting; FBS: fetal bovine serum; FITC: fluorescein isothiocyanate; GFAP: glial fiibrillary acid protein; Glu: glucose; HBSS: Hank's Balanced Salt Solution; H&E: hematoxylin and eosin; HGF: hepatocyte growth factor; HRP: horseradish peroxidase; i.v.: intravenous; MCA: middle cerebral artery; MCH-II: major histocompatibility complex class II; MRI: magnetic resonance imaging; MSC: mesenchymal stem cells; NeuN: Neuronal Nuclei; NF: Neurofilament; OCT: optimal cutting temperature; Olig-2: Oligodendrocyte; PBS: phosphate-buffered saline; PE: phycoerythrin; pMCAO: permanent middle cerebral artery occlusion; PVDF: polyvinylidene fluoride; Pyr: pyruvate; SDS: sodium dodecyl sulfate; STEPS: Stem Cell Therapies as an Emerging Paradigm in Stroke; SVF: stromal vascular fraction; SYP: synaptophysin; TBS: Tris-buffered saline; TBS-T: Tris buffered saline-Tween; TE: echo time; TR: repetition time; TUNEL: terminal deoxynucleotidyl transferase dUTP nick end labeling; VEGF: vascular endothelial growth factor.

## Competing interests

The authors declare that they have no competing interests.

## Authors' contributions

MGF designed the experiments, performed animal experiments, participated in coordination and helped in drafting the manuscript. BRF designed the experiments, performed animal experiments, participated in coordination and helped in drafting the manuscript. JRC and MTVC were responsible for the laboratory assays. BF participated in coordination and helped in drafting the manuscript. SC participed in imaging techniques. EDT designed the experiments, participated in coordination and helped in drafting the manuscript. All authors have read and approved the manuscript for publication.
